# Iron Therapeutics in Women’s Health: Past, Present, and Future

**DOI:** 10.3390/ph13120449

**Published:** 2020-12-08

**Authors:** Joel Mintz, Jackie Mirza, Eric Young, Kyle Bauckman

**Affiliations:** Department of Academic Affairs, Dr. Kiran C. Patel College of Allopathic Medicine, Nova Southeastern University, Davie, FL 33314, USA; jm4719@mynsu.nova.edu (J.M.); jb4382@mynsu.nova.edu (J.M.); ericyoun@mynsu.nova.edu (E.Y.)

**Keywords:** oxidative stress, carcinogenesis, mini-hepcidin, iron therapeutics

## Abstract

Iron plays a unique physiological role in the maintenance of homeostasis and the pathological outcomes of the female reproductive tract. The dual nature of elemental iron has created an evolutionary need to tightly regulate its biological concentration. The female reproductive tract is particularly unique due to the constant cycle of endometrial growth and shedding, in addition to the potential need for iron transfer to a developing fetus. Here, iron regulation is explored in a number of physiologic states including the endometrial lining and placenta. While iron dysregulation is a common characteristic in many women’s health pathologies there is currently a lack of targeted therapeutic options. Traditional iron therapies, including iron replacement and chelation, are common treatment options for gynecological diseases but pose long term negative health consequences; therefore, more targeted interventions directed towards iron regulation have been proposed. Recent findings show potential benefits in a therapeutic focus on ferritin-hepcidin regulation, modulation of reactive oxygen species (ROS), and iron mediated cell death (ferroptosis). These novel therapeutics are the direct result of previous research in iron’s complex signaling pathway and show promise for improved therapy, diagnosis, and prognosis in women’s health.

## 1. Introduction

Iron, an essential transition metal, is integral for the optimal functioning of tissues and organ systems. This is especially true in the female reproductive tract, as women physiologically handle iron differently than their male counterparts [1]. Due to iron’s unique role and function within the women’s reproductive system, it is no surprise that iron dysregulation is noted in many gynecological diseases [2]. Clinical data suggests that the consequences of iron dysregulation can be significant, especially during pregnancy, where the mother is responsible for handling the nutritional requirements for two lives rather than just one [3]. The oldest and most common iron therapy is also the straightforward: iron replacement. Furthermore, clinical findings of iron dysregulation in gynecological disease have led to a number of proposed iron related therapeutics, which operate at different levels of iron homeostasis. Here, we outline the complex interplay of iron, physiology and pathophysiology within the field of women’s health and discuss treatment strategies that target these pathways and their clinical outcomes.

## 2. Iron Chemistry and Metabolism

### 2.1. Iron and Reactive Oxygen Species Signaling

Iron helps maintain homeostasis and regulates a wide variety of physiologic and metabolic pathways, including, oxygen transport, oxidative phosphorylation, and many other enzymatic pathways [4]. Physiologic iron exists in a variety of oxidation states, which determines its biochemistry and cellular actions [5]. In addition to iron’s critical role in energy metabolism and maintaining the production of rapidly dividing tissues like the gastrointestinal epithelium and red blood cells, dysregulation of iron pathways creates reactive byproducts, which causes cellular oxidative stress [6,7]. Oxidative stress is often attributed to generation of hydroxyl radicals and the initiation of lipid peroxidation via the reduction of hydroperoxides present in phospholipids [5,8]. Lipid peroxidation creates mutagenic reactants such as aldehyde malondialdehyde (MDA) [9]. Furthermore, hydroxyl radical generation is not fully understood and may be derived from several sources. However, one commonly reported source of hydroxyl radical production is the Fenton Reaction. This is a process through which Fe^2+^ is oxidized to Fe^3+^ by hydrogen peroxide due to its low oxidation state [8]. Certain environments are more amenable to Fenton chemistry and therefore to the generation of deleterious ROS. The most well studied of these environments is the cytosol, which is why free iron is strictly regulated intracellularly and in tissue beds [10].

### 2.2. Response to Cellular Iron Levels

The iron-regulatory proteins (IRP) IRP-1 and IRP-2 respond to cellular iron levels. Interestingly, the role of IRP is cell specific and includes regulation of proteins involved in iron regulation, tumor suppression, and insulin resistance [11,12,13]. IRP-1′s function varies depending on the concentration of iron. IRP-1 can regulate RNA-binding or catalysis via cytosolic aconitase activity. Aconitase functions in two key biological processes: the citric acid cycle and in iron regulation [14]. IRP-2 exclusively binds RNA at iron-responsive elements (IREs) which are stem-loop secondary structures [14]. Studies suggest that both IRP-1 and IRP-2 bind to the same IRE, which has been demonstrated to modulate the expression of various proteins that further influence iron levels in the body. Some of these proteins include the transmembrane protein ferroportin, which expels cellular iron into the extracellular space, the transferrin receptor-1 (TfR-1), and ferritin, which is utilized for iron storage [5,14]. IRP-2 also possesses a significant role in the development and growth of several cancers. Specifically, overexpression of IRP-2 has been implicated in the suppression of TAp63, which is a member of the p53 tumor suppressor gene family [15]. Takenaka et al. have observed that under hypoxic conditions often experienced by tumor cells, IRP within ovarian cysts is stabilized, which contributes to the malignant transformation of ovarian cysts into tumor cells [16].

### 2.3. Ferritin and Iron Metabolism

A key factor in the regulation of iron is the peptide hormone hepcidin, which is primarily synthesized in the liver. Hepcidin expression is primarily regulated at the level of transcription, and this process is influenced by such factors as the presence of inflammatory mediators, like IL-6, and the availability of free iron [17,18]. The production of cytokines in the settling of inflammation is thought to modulate the expression of hepcidin via the JAK-STAT3 inflammatory pathway [18]. Therefore, binding to the promoter region of the hepcidin gene is a primary target for the modulation of its expression [18]. Additionally, studies have suggested that tumor suppressors, such as p53, bind and activate the hepcidin promoter region, thus providing an explanation for the increased hepcidin production in the context of carcinogenesis [19]. Although a number of regulatory mechanisms that control hepcidin synthesis and function have been proposed, the end result is decreased iron availability by most cell types in the body [20]. 

**Figure 1:**
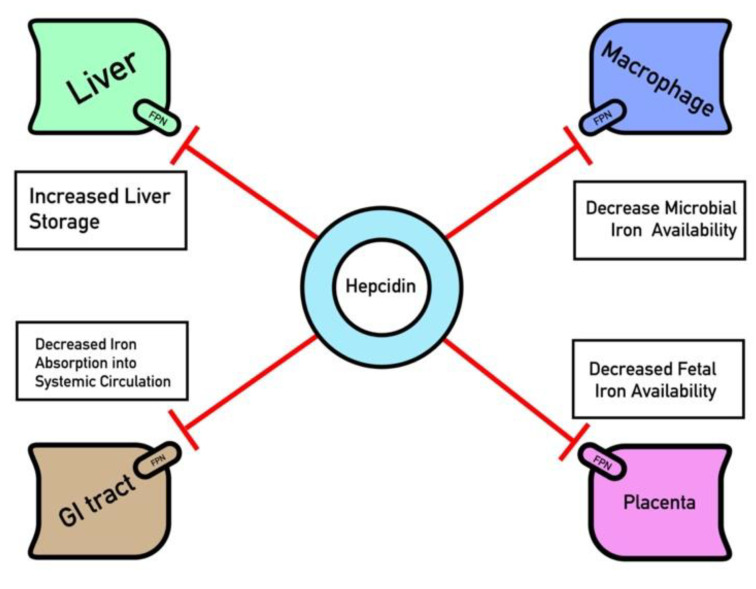
Ferroportin (FPN) regulation by Hepcidin. Key organ systems contain ferroportin channels that are degraded by hepcidin such as the GI epithelium, reticuloendothelial macrophages, hepatocytes and placenta. Hepcidin is up-regulated in response to hyperfermia and inflammation, which serves to sequester iron within these tissues and decrease iron availability.

Once synthesized, hepcidin regulates iron in the body primarily via binding to ferroportin on the membrane of iron-exporting cells, which subsequently causes ferroportin to be endocytosed (Figure 1). Such cell types are thought to traditionally include hepatocytes, reticuloendothelial macrophages, and GI epithelium cells, although other cell types are also thought to undergo hepcidin regulation, such as those that comprise the blood brain barrier [20,21]. Following endocytosis, ferroportin is ubiquitinated and proteolyzed [20]. Although iron can enter the cell through the unidirectional divalent metal transporter-1 (DMT-1), the destruction of ferroportin traps iron bound to ferritin inside cells, preventing release into systemic circulation [20,22]. This process is critical during states of iron overload, where high levels of circulating free iron can damage cells [23]. Due to hepcidin’s integral role in iron homeostasis, its dysregulation has been implicated in both hypoferremic states as anemia of chronic disease and hyperferremic states as hemochromatosis [24,25].

Furthermore, iron sequestration is an innate defense against invading microorganisms, and hepcidin is notably upregulated in the presence of pro-inflammatory mediators [26]. Similar to humans, iron is also essential for the replication of exogenous pathogens causing infection [27]. Due to this competitive interplay, iron sequestration in humans is a critical defense mechanism against foreign pathogens. Hepcidin has antimicrobial properties and is sometimes referred to as LEAP (liver expressed antimicrobial peptide) due to its ability to induce hypoferremia [27,28]. Patients with iron-overload, like those with hemochromatosis, have an increased susceptibility to infection due to their hyperferremic state with a high availability of iron [27]. Thus, hepcidin has an important function as an acute phase reactant [29]. Furthermore, the generation of pro-inflammatory cytokines, such as interleukin-6, interferon-γ, and TGF-β, in the setting of infection encourages the production of acute phase reactants, such as hepcidin, which then downregulates serum iron concentration and availability [30,31]. For instance, both hepcidin and ferritin are upregulated in the setting of inflammation, whereas transferrin is downregulated. In total, all work together to effectively increase iron scavenging and decrease the available iron for pathogen propagation [32]. 

## 3. Physiological Need for Iron Handling within the Women’s Reproductive System

### 3.1. Endometrial Shedding and Menses

Iron is required for all rapidly dividing tissues, and the endometrium is no exception [33]. Similar to other cell types, endometrial tissues highly express DMT-1, which is known to transport iron across the cell membrane for storage as ferritin [34,35]. Free ion concentration inside the endometrium must be carefully regulated, as excess iron inside the endometrium can increase the production of tissue damaging ROS [36]. Increased expression of DMT-1 is noted amongst many patients with endometriosis, and ROS produced through iron dysregulation is thought to potentiate pathogenesis [34]. A study of rats suggested that endometrial stromal cells can differentiate into highly phagocytic iron storing cells, which the authors hypothesize function to digest secreted glycoproteins and have lipofuscin storage ability [37]. However, this hypothesis has not been tested in humans and rats contain more stromal cells and less glandular tissue [37]. Multiple evolutionary hypotheses have been proposed that postulate the need for menstrual bleeding, although the environmental selection pressures behind menstruation remain controversial and under study [38]. Whatever the underlying evolutionary mechanism for the development of menstruation, iron is lost through each menstrual cycle through excretion of endometrial cells and iron rich hemoglobin, which can cause clinically significant anemia in women with heavy menstrual bleeding (Table 1).

### 3.2. Iron Handling across the Placenta

**Figure 2:**
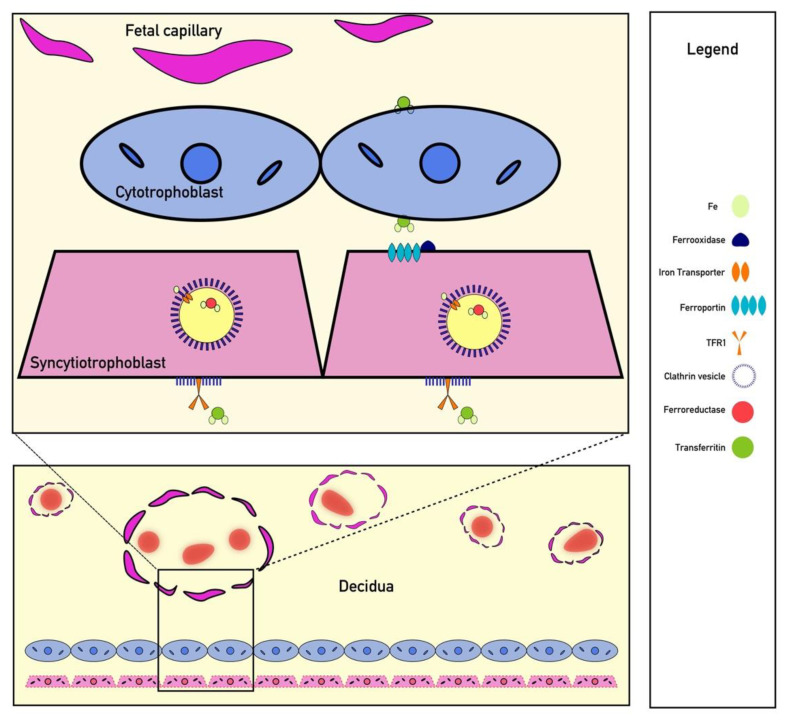
Iron Transport Across the Placenta. Iron is transported across the placenta by bind to TFR1 receptors on syncytiotrophoblast surrounded by clathrin-coated pits. Endocytosis of these receptors is followed by ferroportin transport. Iron is ultimately shuttled to fetal capillaries by transferrin.

Iron is a critically important micronutrient for proper fetal development; however, iron cannot easily diffuse through the placenta to reach the fetus [68]. Additionally, the fetus cannot create its own iron and must receive all of its iron from the material circulation [69]. Circulating iron is often bound to transferrin, and transferrin-bound iron uptake appears to be necessary for fetal development [69]. One study of iron transport found that embryos of transferrin receptor-1 (TfR1) knockouts were not compatible with life due to severe anemia [69,70]. Once transferrin-bound iron binds to TfR1 on syncytiotrophoblasts, clathrin-mediated endocytosis occurs, and iron is released from transferrin within the acidified endosome [71] (Figure 2). From there, iron is reduced from Fe^+3^ to Fe^+2^ via ferrireductase and exported into the cytoplasm via DMT-1 or ZRT/IRT-like protein (ZIP) before being bound to an iron chaperone for transport to ferroportin, which faces the fetal basal lamina connective tissue [69]. Iron transport across the placenta results in iron binding to fetal transferrin, although the mechanism by which iron is transported from the basal lamina connective tissue into the fetal circulation remains under study [69]. Other mechanisms related to non-transferrin bound iron (NTBI) placental transport have been proposed, but their significance in placental development is currently unknown [69]. Importantly, NTBI appears only in iron overloaded patients with significantly elevated transferrin saturation and is unlikely to play a significant role in physiological placental iron transfer [69,72]. Recently published evidence suggests that hepcidin also functions within the placenta to downregulate ferroportin and TfR1 expression [42]. Therefore, systemic conditions that affect hepcidin concentrations should also be assumed to modulate iron transfer to the fetus in a similar mechanism to other tissues. However, more preclinical research is needed to directly prove that hepcidin is the direct cause of iron dysregulation noted in many of the observational clinical studies discussed elsewhere.

### 3.3. Iron Handling during Pregnancy and Lactation

Several maternal and fetal mechanisms increase iron metabolism during pregnancy. Daily iron requirements in pregnancy accelerate from 0.8 mg/day to 7.5 mg/day during the latter trimesters, as fetal growth quickens [73]. Maternally, iron requirements are driven in large part by blood volume expansion, placental development, and typical enterocyte and epidermal cell turnover. Each function is estimated to require 450, 90, and 230 mg of iron, respectively [74]. Iron consumption due to fetal development is approximately 270 mg, and total iron needs exceed 1 g per pregnancy for an estimated average of 4.4 mg per each day of pregnancy [73,74]. To accomplish the growing iron requirements in the second and third trimesters, hepcidin is significantly downregulated, which increases systemic iron absorption and release [74]. In rats, pregnancy reduced transferrin saturation and increased the expression of multiple iron transporters throughout the gastrointestinal tract and body, suggesting increased iron mobilization to support the growing fetus [75]. Although the underlying mechanism that inhibits hepcidin remains unknown, hepcidin levels return to baseline shortly after fetal delivery [74]. One potential mechanism for hepcidin’s return to baseline is RBC mass contraction after delivery increases salvaged iron stores [74]. Therefore, hepcidin is upregulated to inhibit iron absorption and drive iron storage as ferritin, preventing iron overload [74]. Other serum markers of iron handling also decrease throughout pregnancy and then increase postpartum, including transferrin, which results from a combination of increased iron handling and hemodilution due to blood volume expansion [74,76]. Pregnancy is characterized by expansion of both RBC mass and volume to accommodate placental and fetal growth. Notably, volume increases more than mass, resulting in lower hematocrit and hemoglobin concentration [77]. Such physiological changes can have a wide variety of clinical effects, and iron supplementation is almost universally recommended for pregnant women [78].

After birth, a major source of infant nutrition is that of breast milk. However, human breast milk is thought to contain less iron than is required to meet an infant’s nutritional needs, as human breast milk has a median iron concentration of 0.47 mg/L (0.04–1.92 mg/L) [79]. Interestingly, the concentration of iron in breast milk is independent of maternal factors, including iron reserves, dietary iron intake and other environmental or behavioral factors [79]. Despite the low iron content in human breast milk, healthy infants are suggested not to need iron supplementation and are at little risk for iron deficiency while consuming breast milk [79].

### 3.4. Disruptions to Iron Handling during Pregnancy

Physiological iron transfer during pregnancy can be disrupted by a number of physiological and environmental factors. One such environmental factor is tobacco smoke, which is thought to suppress hepcidin through systemic hypoxia [39,40]. A similar effect on hepcidin synthesis was noted in pregnant women who smoked and the degree of hepcidin suppression was linearly correlated to the number of cigarettes smoked per day [41]. However, serum hemoglobin was higher, and iron parameters were lower in the smoking cohort [41]. These findings suggest that iron transfer to the fetus may actually be compromised, despite hepcidin suppression, as more iron is shuttled to make red blood cells to combat the systemic hypoxia induced by cigarette smoking. Thus, impaired iron transfer may also be a causal factor of low birthweight amongst women who smoke, in addition to intrauterine toxin exposure and other mechanisms [80]. Studies suggest that iron metabolism and transfer during pregnancy is multifactorial. For example in mothers with intrauterine growth restriction (IUGR), hepcidin concentration was similar to that of matched controls [43]. However, ferritin expression was lower in the cord blood of mothers with IUGR suggestive of impaired iron placental iron transfer, which may be caused by hepcidin induced downregulation of placenta ferroportin due to inflammation, a characteristic finding of IUGR [43,44]. When these results are combined with other studies that suggest iron deficiency anemia is a risk factor for low birth weight, it is clear that reduced placental iron transfer may reduce fetal growth [81].

However, not all adverse conditions during pregnancy involve reductions in serum iron parameters. For example, in preeclampsia, serum iron parameters are increased, in spite of increases in hepcidin [82,83]. Although the exact mechanism underlying the elevation in iron remains unknown, researchers have proposed that increased absorption of dietary iron combined with inappropriate excess iron supplementation may actually be causal for the disorder [45]. Increased serum iron early in pregnancy has been proposed to set off a cascade of irreversible iron-related adverse reactions when the spiral arteries cannulize and oxygenated blood begins to flow through the placenta [45]. Potentially indicated processes include iron mediated hypoxia-reperfusion injury, ferroptosis, mitochondrial dysfunction, and aberrant lipid peroxidation [45]. It is unknown exactly how elevations in serum iron and hepcidin affect iron transfer in preeclampsia; however, hepcidin downregulates placental ferroportin independent of serum iron, suggesting that placental iron transfer may actually be restricted, in spite of high serum iron [42]. For women with gestational diabetes, umbilical cord blood showed an increase in umbilical cord ferritin and ferroportin, which indicates increased iron transfer to the fetus [46]. Macrosomia, a notable complication of gestational diabetes, was also shown to be independently related to high ferritin concentrations, suggesting that increased iron transfer to the fetus may be crucial in the pathogenesis of macrosomia [47,48]. However, it is not currently known if this is a causal mechanism or a byproduct of increased growth. Nevertheless, the growing body of literature discussing iron handling in pathological obstetric conditions provides firm evidence that impaired iron transfer to the fetus during pregnancy can be a risk factor for disruption to the healthy growth and development of the growing fetus.

### 3.5. Iron and Genitourinary Tract Infections

Certain pathogens, like the uropathic *Escherichia coli* (UPEC)*,* have evolved to circumvent host cells’ iron sequestration response. Recent studies have demonstrated that ferritinophagy, a process that releases iron from its ferritin-bound state in host cell lysosomes, facilitates the propagation of UPEC by increasing the available iron for bacterial replication [49]. The clinical significance of this discovery lies in its potential for treatment of urinary tract infection (UTI), a pathology with a self-reported 12% annual incidence in all women [84]. Treatments targeting the processes of ferritinophagy, iron chelation therapies, and even the dietary restriction of iron show some promise in helping treat UTIs [49,85,86].

In addition to UTI’s, infectious vaginal processes may also be influenced by local microenvironmental factors, including iron. It is currently unclear if this process is related to proliferation of siderophore producing microorganisms within the vaginal tract or some other mechanism, as increases in soluble transferrin receptor (sTfR) was noted in patients with bacterial vaginosis during pregnancy (BV) [50]. sTfR was positively correlated to the presence of bacterial vaginosis, while serum iron parameters were not, suggesting iron deficiency may facilitate the propagation of BV [50]. Although the mechanism underlying this finding is unknown, the function of iron within cellular defense and infection prevention is complex and may differ depending on the location and type of tissue infected [50,85,86]. Therefore, both identifying and targeting factors that affect the growth of these pathogenic flora provide a potential opportunity for future iron mediated therapies [87]. Potential treatments targeting pathogenic iron usage by microbes will often focus on the process of pathogen iron acquisition either by binding siderophore directly or by modulation of siderophilic pathways [27]. Additionally, a new promising avenue for the treatment of infectious disease is the development of novel vaccines that target classes of molecules involved in iron acquisition via outer membrane receptors [88].

## 4. Traditional Iron Therapeutics: Modulation of Biological Iron

### 4.1. Abnormal Menstrual Bleeding

In addition to the physiological necessity of iron within all rapidly dividing tissue such as the endometrium, blood loss during menses can also affect total body iron stores [33]. Early clinical studies found that over 35 mL of blood is lost on average per menstrual cycle; however, the amount of blood loss is highly variable [89]. Typical menses last approximately 5 days. In some patients with longer cycles who were not taking supplemental iron, ferritin levels were lower, suggesting that heavy menstruation can deplete total body iron stores [90]. Although 1 mL of packed red blood cells contains approximately 1 mg of iron, approximately 1 mg of iron is lost each cycle in women with normal menstrual bleeding which usually does not deplete iron [91,92]. Serum iron usually remains unchanged unless the bleeding is severe, and an iron deficiency occurs (>80 mL) [93]. However, measuring blood loss during menstruation is difficult, and a clinical diagnosis of heavy menstrual bleeding requires the bleeding to be severe and interfere with functioning [51]. Importantly, a clinically significant anemia may not always occur in patients with heavy menstrual bleeding, as a complete blood count alone had less than 50% sensitivity and specificity at diagnosing iron deficiency [94]. Iron loss due to menstruation remains a significant clinical problem as almost half of all heavy menstrual bleeding patients display an iron deficiency or have iron deficiency anemia [51]. Additionally, heavy menstrual bleeding accounted for over 50% of all recognized cases of iron deficiency anemia [51]. Heavy menstrual bleeding adversely impacts biopsychosocial functioning and treatment of an underlying iron deficiency can potentially mitigate the psychosocial effects of heavy menstrual bleeding and improve quality of life [95,96].

Iron replacement is used therapeutically in women who are iron deficient due to heavy menstrual bleeding and often occur alongside hormonal contraceptive treatment, which is considered first line therapy [51,97]. Iron replacement can be administered orally or intravenously and has a number of different formulations, although oral iron sulfate is commonly used [98]. Heavy menstrual bleeding can occur in a number of pathological obstetric and hematologic conditions, so concurrent investigation into other possible underlying causes should be performed in addition to correction of the underlying iron deficiency or anemia [51,97]. Dosing of iron replacement depends upon the severity of the underlying deficiency and the clinical status of the patient. Approximately two thirds of inpatient admissions for heavy menstrual bleeding were found to need a therapeutic blood transfusion by one study, although the underlying iron deficiency may be more severe in inpatient units and baseline iron parameters were not measured [99]. Therapeutic blood transfusion is not without a significant side effect profile and intravenous iron is generally well-tolerated [98]. For heavy menstrual bleeding due to an underlying pathological lesion, 500–1500 mg of intravenous ferric carboxymaltose was used with considerable efficacy, as hemoglobin generally improved 2 g/dL or more while ferritin improved over 100 ng/dL [100]. Parenteral iron can potentially replete iron stores in a single dose lowering the number of physician visits, however patient costs may be greater than oral therapy [98]. To determine the total iron needed to make the patient iron replete the following formula can be used: weight (kg) × 2.3 × (target hemoglobin—current hemoglobin) + either 500 or 1000 mg depending upon severity [98]. Intravenous iron side effect profiles consist of transient nausea, flushing, or headache in addition to rare hypersensitivity or allergic reactions [98].

For patients with less severe iron deficiency, oral therapy is often considered (Table 2) [101]. Randomized control studies found that gastrointestinal iron absorption was decreased with daily oral iron therapy and was due to increased hepcidin which provided negative feedback on iron absorption [102,103]. Typically, a 65–135 mg once daily oral dose of iron sulfate is used to correct iron deficiency anemia and aid in compliance; however, in light of previously discussed evidence, it may be beneficial to conduct clinical studies which examine modifying treatment regimens to once every other day [97]. Oral iron therapy is associated with gastrointestinal side effects, which can be minimized by evening dosing with food consumption [97]. Heavy menstrual bleeding can have a wide array of underlying etiologies and effects, which necessitates the need for patient specific management. Furthermore, the risk and benefits of iron replacement therapy should be discussed with patients before initiating therapy.

### 4.2. Iron Deficiency and Iron Deficiency Anemia of Pregnancy

Physiological iron balance is disrupted in pregnancy as iron is shuttled to the placenta and fetus for growth [74]. Iron deficiency during pregnancy is common, as one study found that over 90% of all pregnant women are iron deficient [104]. The prevalence of those who experience a clinically significant anemia as a result of iron deficiency varies, with an estimated prevalence of 40%, although 20% of all pregnant women may experience a hemoglobin of less than 8 g/dL [52,105]. The prevalence of iron deficiency anemia grows to over 50% in regions where nutritional deficiencies and food scarcity are common, such as Sub-Saharan Africa [106].

Generally, as more iron is transferred to the fetus for growth, iron stores are mobilized from reserves, depleting iron total body iron [52,53]. Less iron is then able to be transferred to precursor RBCs affecting erythropoiesis [107]. Over time, healthy RBCs in circulation are recycled and replaced with their hypochromic and mircocytic counterparts, which can result in anemia if iron is not supplemented or oral intake does not increase [52,107].

The consequences of disruptions to iron metabolism during pregnancy are significant. Iron deficiency in the first trimester and iron deficiency anemia in general during pregnancy are associated with lower birth weights and increased risk of preterm labor, although not all studies support these findings. [52,53,108]. Furthermore, earlier diagnosis of iron deficiency anemia in pregnancy was associated with elevated risk of preterm birth, and a few retrospective studies have identified anemia as increasing maternal mortality especially for hemoglobin levels under 8 g/dL [52,53]. Importantly, iron deficiency carries a consequence for the infant as well, as one study of half a million children found a significantly increased risk of neurodevelopmental disorders in children who were born to mothers who experienced anemia before 30 weeks of gestation [109]. This effect is hypothesized to be multifactorial, as pre-term birth and adverse events during pregnancy can cause developmental delays [110]. However, iron deficiency anemia during pregnancy has been associated with iron deficiency during the first year of life, which may also cause developmental delays [111]. Diagnosis of iron deficiency anemia of pregnancy may be confirmed by finding a serum ferritin of <15 ug/dL [52]. However, ferritin is an acute phase reactant, and serum ferritin may not always reflect iron stores, especially peri- and postpartum or in the presence of an infection [52].

Increasing iron intake by 15–30 mg through diet or supplementation is recommended for most pregnant women, and iron is commonly formulated into many prenatal vitamins [52]. Hemoglobin below 10 g/dL is often defined as iron deficiency anemia of pregnancy, and this threshold was most likely to be met around 25 weeks [112]. First and third trimester hemoglobin below 11.0 g/dL is also worrisome for iron deficiency anemia [52]. Evidence supporting treatment of mild anemia in pregnancy is mixed and despite a number of studies showing improvement in hematological parameters, few measure clinical outcomes [113]. However, treatment is often initiated as the patient’s clinical course can rapidly decline in some cases [52] Therapeutically, oral iron is considered the gold standard therapy for mild to moderate anemia, which can be corrected with an additional oral dose of 30 to 120 mg [52]. Generally, iron supplements are dosed between 100–200 mg and should be taken with vitamin C to increase absorption [51,98]. The clinical effect of iron supplementation is most apparent after 25 weeks of gestation, where serum hemoglobin and iron parameters trend upward for those receiving iron supplements [53,112]. Daily iron supplementation is especially important as the mother approaches term, as daily iron supplementation lowered anemia and iron deficiency by 70 and 50% respectively [52]. Gastrointestinal side effects of oral iron supplementation appear to be particularly prevalent amongst pregnant women and are a major limiting factor affecting therapy adherence [51]. One large multicenter study found that intravenous ferric carboxymaltose may improve hemoglobin and iron repletion more than oral preparations, in addition to improved socio-behavioral outcomes and reduced gastrointestinal side effects [114]. While the clinical benefit remains unclear, it may be useful for rapid iron correction, rather than preemptive supplementation [114]. Deficiencies of folate and other vitamin deficiencies are also common culprits of anemia in pregnancy [112]. Therefore, laboratory workup for other vitamin deficiencies should also be considered when evaluating the anemia’s underlying cause [112].

### 4.3. General Iron Supplementation and Iron’s Role in General Health

Iron supplementation amongst non-pregnant, non-lactating, menstruating women, suggest that iron supplementation can correct iron deficiency, improve ferritin levels, and replete total body iron; however, the clinical outcomes of this finding in the general population are unclear [90]. Special populations of women, namely athletes, may be at an increased risk of iron deficiency, although neither in-season competition nor iron supplementation had any effect on iron status [115,116,117]. General iron supplementation is of questionable benefit in elderly women, and one study on dietary supplements found increased all-cause mortality with general iron supplementation that was exacerbated by higher dosage [54]. Increased dietary hemoglobin iron is associated with elevated risk of coronary artery disease, cardiovascular mortality, and colon cancer, while supplemental and hemoglobin iron were associated with an increased risk of type 2 diabetes mellitus amongst that same cohort of women [55,56,57,58]. Menopause removes one physiological means of excreting iron, and iron levels are notably higher in women with menopause [59]. Furthermore, links between iron and other pathophysiologic menopausal features have been identified such as hot flashes, skin photoaging and osteoporosis [59]. Cessation of menstruation in amenorrheic states is also associated with higher iron stores, which was demonstrated by lower levels of iron reserves in menstruating exercising women when compared to other women in a similar cohort of women who had amenorrhea [118].

### 4.4. Iron Replacement for Gynecological Anemia of Chronic Disease

Many gynecological and other cancers can cause anemia of chronic disease, whereby circulating inflammatory cytokines released in response to the tumor may force iron suppression [60]. Restoring serum iron through intravenous administration may also improve symptoms and reduce complications. For gynecological cancers, IV administration of iron sucrose lowered the number of transfusions needed after receiving chemotherapy [60]. IV administration of iron can improve symptoms, hematological labs, and quality of life; however, oral administration is inefficacious for treating malignancy induced fatigue [60]. Alternatives include transfusion and erythropoiesis-stimulating agents (ESA); however, such treatments also confer an increased risk of kidney injuries, transfusion reactions, and thrombolysis. Although transfusion can be used to mitigate severe symptomatic anemia, ESAs may only benefit a minority of patients [119]. Clinical guidelines surrounding iron supplementation for anemia of chronic disease remain ambiguous and treatment decisions are often left to the provider, although rigorously monitoring of iron labs may be required [119].

### 4.5. Iron Replacement in Other Conditions

A commonly reported symptom of iron deficiency anemia is fatigue. Complaints of fatigue should prompt investigation via a complete blood count to rule out anemia. Importantly, for unexplained fatigue, treatment with 800 mg of intravenous iron (III)-hydroxide sucrose improved fatigue in women with serum ferritin under 15 mg/dL [120]. Furthermore, iron supplementation also decreased muscle fatigability during exercise within iron depleted non-anemic women [121].

### 4.6. Iron Overload and Chelation in Women’s Health

Although iron deficiency anemia of pregnancy is common, there is no clear association with clinical outcomes [122]. Therefore, some propose that supplemental iron in iron-replete women actually place pregnant women at risk of iron toxicity [45]. Iron toxicity has much of the same effects as iron deficiency and places the fetus at risk of preterm birth and low birth weight [45]. Furthermore, hypertensive disorders of pregnancy are associated with high iron levels and pre-eclampsia has been proposed to result from ferroptosis induced via iron overload [45]. Serum MDA is also highly elevated in preeclampsia further suggesting iron dysregulation [45]. However, iron chelation that reduces biological iron is still relatively rare in women’s health. Iron chelation is considered during pregnancy in women with thalassemia, as a transfusion may aggravate iron overload and increase oxidative stress [123]. Oral iron chelators are typically not considered, because they do not cross the placenta [123]. Development of cardiac symptoms or ventricular dysfunction should prompt consideration of chelation therapy with deferoxamine, although deferoxamine is typically avoided in the first trimester due to unknown effects on fetal development [123,124]. However, case reports and small case series describe good outcomes after treatment with deferoxamine in pregnant women with thalassemia [123].

## 5. Modern Iron Therapeutics: Reducing Oxidative Stress and Carcinogenesis

### 5.1. Oxidative Stress Targeting in Endometriosis and Other Gynecological Diseases

Various oxidative stress models and pathways have been proposed for the pathogenesis of many gynecological diseases. ROS serve as a vital target for iron-based treatments due to the amenability of iron to be reduced via Fenton chemistry and generate harmful ROS [10]. Previous studies have demonstrated elevated levels of iron, hemoglobin, and oxidative stress markers in women with endometriosis, especially in endometrial cysts [61,62].

Not only does oxidative stress harbor mutagenic potential, but aberrantly generated ROS may also have a role in the modulation of cell proliferation [61], which may lead to endometriosis [61]. Additionally, Huixia, L. et al. proposed a mechanism that suggests reactive oxygen species (ROS) induce autophagy of ectopic endometriosis cells with antioxidants N-acetyl-L-cysteine and catalase reducing markers of autophagy [63]. Several pro-inflammatory markers have been identified in ectopic endometrial lesions. Upregulation of the enzyme amine oxidase 3 (AOC3) may contribute to the oxidative stress within endometriosis as in vivo studies indicate that inhibition of AOC3 may provide analgesic effects in endometriosis [125]. Furthermore, one study evaluated the role of haptoglobin in endometriosis and determined that haptoglobin and IL-6 secreted by endometriosis cells decreased the adherence of peritoneal macrophages to the endometrial lesions and contributed to the pathophysiology of endometriosis [126]. Together, these studies suggest that iron dysregulation is central to the pathophysiology of endometriosis

Potential iron therapeutics that function by reducing the number of reactive oxygen species generated from iron dysregulation and inflammation have been proposed to combat the growth of endometrial lesions. For example, melatonin demonstrates radical scavenger capabilities, and preclinical studies suggest melatonin may decrease the size of ectopic endometriosis lesions [127]. Furthermore, one randomized clinical trial demonstrated that melatonin can be used as an antioxidant and is capable of reducing chronic pelvic pain in endometriosis, making melatonin a potential adjunctive treatment option for women suffering from endometriosis [128]. N-acetyl-cysteine (NAc) has demonstrated anti-inflammatory and antioxidant properties useful for the treatment of endometriosis, and NAc may also reduce the lesion size of endometriosis lesions [129]. Additionally, NAc therapy has limited side effects and teratogenicity, making NAc potentially suitable for treating symptoms of endometriosis in pregnant women, although clinical studies are needed to confirm therapeutic benefit [130]. Other antioxidants such as vitamins C and E have been assessed in clinical trials for treating endometriosis. These antioxidants have demonstrated reduction in pain symptoms and inflammatory markers [128]. Some natural antioxidants which have been studied in endometriosis include flavanones such as apigenin and luteolin, although their clinical efficacy remains under study [131,132]. Current research continues to focus on reducing agents and antioxidants that either target the oxidative stress or induce cytotoxicity to endometriosis cells.

Others have proposed the usage of iron related therapies targeting oxidative stress at the level of mRNA. For example, one study demonstrated that microRNA miR-455 is involved in targeting and downregulating the protein fatty acid binding protein 4 (FAB4), which is implicated in inducing oxidative stress in endometriosis cells [133]. Another study demonstrated efficacy of the reducing agents glutathione and cysteine complexed with binuclear dinotrosyl iron complexes (DNIC) in the reduction of the size of endometriosis tumors in surgically-induced endometriosis mice models [134].

### 5.2. Carcinogenesis

Iron dysregulation is suggested to be able to potentiate carcinogenesis after studies noted the presence of DNA damage and oxidation byproducts in cancer tissues [9]. DNA damage and modification, especially via the formation of DNA adducts, is the cornerstone of mutagenesis and by extension, carcinogenesis. Due to the predilection for free radicals to modify the nitrogenous base, guanine, the presence of 9-hydroxyguanine (8-OH-G) is sometimes used as a means of risk assessment and to further inform on potential past exposure to carcinogens, although its use as a diagnostic measure requires further research [5].

Paradoxically, many experimental anti-cancer therapeutics involve the chelation of iron due to its utility in DNA synthesis and, by extension, cell proliferation [135]. Malignant tumors often have rapid rates of cellular division and have a higher metabolic demand for iron [136]. Decreased availability of iron for uptake by chelating agents such as Deferoxamine (DFO) has been proposed as antineoplastic agents [137]. The facilitation of the increased uptake of iron by neoplastic cells has been explained by an increase in the expression of the cell surface transferrin receptor TfR1, an iron import protein [138]. This increase in TfR1 expression may be especially prominent when considered following the administration of chelators due to decrease available iron for uptake. Nucleic acid-based therapeutics targeting TfR1 mRNA show promise especially in their specificity to cancerous cells [139]. Amenability to such targeted treatments may be assessed by using proteomic analyses to determine TfR1 expression in individuals.

Given the high rates of resistance to chemotherapy that often develop in cases of ovarian cancer, identifying new targets for treatment is of vital importance. Resistance to traditional antineoplastic treatments has been attributed to the presence of multipotent cells that have been termed cancer stem cells [140]. Identification, and obliteration, of these cells represent an avenue for treatment that is less likely to develop resistance. Tfr1 as a therapeutic target shows promise in ovarian cancer due to previous studies demonstrating an increased expression of Tfr1 receptors in malignant ovarian cancer cells as compared with non-malignant tissue [141], in addition to increased intracellular iron stores in neoplastic cells. Therefore, targeting the Tfr1 receptor may be a useful mean to reduce iron availability of iron for use by cancerous ovarian cells.

## 6. Future Directions: Targeting Iron Homeostasis through Hepcidin Therapeutics

### 6.1. Hepcidin as a Diagnostic Biomarker

Hepcidin plays a central role in maintaining iron homeostasis and represents a promising frontier for the diagnosis and treatment of many conditions, including those in women’s health [142,143]. Diagnostically, hepcidin is particularly useful as a prognostic biomarker for tumors of the ovary and breast [65,144]. Furthermore, hepcidin levels are correlated to total body iron stores, insulin resistance in polycystic ovarian syndrome, gestational diabetes mellitus, and general and pregnancy induced iron deficiency anemia [145,146,147,148,149,150]. Furthermore, hepcidin levels were correlated to preeclampsia and associated adverse fetal outcomes including low birth weight and NICU admission [151]. However, many of these studies were conducted in resource poor settings, and the effect of diagnostic hepcidin on clinical decision making as well as the cost-benefit analysis of implementing diagnostic hepcidin remains poorly understood. Nevertheless, asymptomatic iron dysregulation can precede the development of other diseases, such as Huntington’s [152]. Although it is currently unknown if iron dysregulation also similarly precedes gynecological and obstetric diseases, future clinical studies should examine if hepcidin dysregulation can be used to detect disease onset prior to the development of symptoms or improve patient risk stratification models.

### 6.2. Hepcidin Therapy

Generally, hepcidin therapies are divided into direct agonists and antagonists, although regulation of hepcidin through JAK/STAT3, BMP/SMAD, IL-6 and sex hormone signaling have also been explored [153]. Sex hormone therapy may have the greatest relevance to women’s health, as one study found that endogenously produced estrogen drastically decreased hepcidin levels over three fold, suggesting reduced iron suppression [154]. Interestingly, oral contraceptives are pharmacotherapy for women with abnormal menstrual bleeding [155]. Therefore, oral contraceptives may have a dual mechanism in preventing iron deficiency and iron deficiency anemia in women with heavy menstrual bleeding. First, reduction of bleeding prevents iron loss, and second, increased estrogen may inhibit hepcidin allowing increased iron uptake and absorption [156]. However, the efficacy of oral contraceptives as stand-alone agents that can prevent iron deficiency is questionable. Clinical evidence suggests that in a subgroup analysis of over 14,000 women, oral contraceptives had no effect on the rates of iron deficiency [157]. Further complicating the use of oral contraceptives as hepcidin modulators are other studies that suggest in older, post-menopausal women hormone replacement therapy reduces ferritin levels. There is clear evidence suggesting that exogenous sex hormone therapy modulates iron levels, although future work is needed to determine if hepcidin modulation provided by these agents is of direct clinical benefit.

Direct or indirect antagonists of hepcidin may increase iron availability and have potential therapeutic uses for conditions in which iron availability is low. For example, hepcidin antagonists may be able to increase dietary iron absorption and release stored which may help correct anemia due to many causes including pregnancy, gynecological cancers, and menstrual bleeding. Inflammatory obstetric disorders where hepcidin levels are elevated may also benefit from direct hepcidin antagonists, particularly those with IUGR as elevated hepcidin released from inflammation at the endometrium suppresses iron uptake, further restricting fetal growth. Complicating the potential therapeutic applications of direct hepcidin modulators are the limited preclinical studies examining teratogenicity of such compounds. Without such studies, it is difficult for researchers to reach the high but necessary bar to perform clinical studies on pregnant women, long considered a protected patient population for human subjects research. Current clinical trials of hepcidin antagonism are primarily centered around malignancy induced anemia of chronic disease and other hematological disorders, although quinoxaline, a small molecule that prevents degradation of the ferroportin receptor was shown to have in vitro activity against the MCF7 breast cancer cell line [153,158]. Previous work has shown that low hepcidin levels in breast cancer cell lines was of particularly favorable prognosis, which may be related to the concentration of intracellular iron and the creation of ROS, driving cancer progression [65,66]. Furthermore, disruption of intracellular iron signaling also has been shown a useful adjuvant agent to traditional chemotherapy for ovarian and breast cancer [159]. Although limited evidence exists on the effectiveness of hepcidin antagonists in gynecological cancers, intracellular iron is required for the division of all malignant tissues [67]. Furthermore, elevated levels of hepcidin were noted in endometrial lesions, suggesting that ferroportin down regulation prohibits iron excretion out of endometrial cells and can ultimately lead to increased oxidative stress [64]. These findings suggest that the most promising avenue of anti-hepcidin therapy is for benign and malignant gynecological tumors.

Currently, clinical studies of hepcidin agonists primarily center around the use of direct hepcidin formulations, although their uses are currently limited to iron overload induced by hereditary hemochromatosis and beta-thalassemia [153]. However, natural hepcidin is limited by its short half-life, several synthetic methods of mini-hepcidin are aiming to increase the half and therefore the beneficial therapeutic effects similar to the role of hepcidin [160,161,162]. These mini-hepcidins consist of a short chain of amino acids that mimic the N-terminal region, the ferroportin binding region [153]. To date, no preclinical studies of mini-hepcidins have a women-centric health focus, although hepcidin is thought to function heavily in antibacterial defense of the urinary tract [153,163]. However, it is currently unknown if potential hepcidin agonism can be used to aid host defenses, especially in women with recurrent UTIs. Furthermore, obstetric disorders where there is evidence of iron overload, such as gestational diabetes mellitus, may also benefit from the utilization of these therapies, although they suffer from many of the same limitations regarding teratogenicity as their antagonistic counterparts. Similarly, hepcidin agonists may also be useful prior to the onset of preeclampsia as iron overload is thought to be central to pathogenesis; however, women remain asymptomatic during the early stages, and after the onset of symptoms, hepcidin is upregulated. Therefore, until a way to screen for the disease is developed, hepcidin agonists may be limited for this purpose. Although clinical studies have yet to define the exact role of hepcidin agonism within women’s health, the potential for uses of hepcidin agonists appears to be more limited than that of hepcidin antagonists, as low iron availability, excess iron storage, or reduced placental transfer of iron appear to be implicated.

## 7. Conclusions

Iron plays a unique role in women’s health due to its unique physiological function within women’s health. Excess iron may contribute to oxidative stress and pathology within women’s health, and therefore, iron levels must be tightly regulated. There is an essential need for iron during menstruation and pregnancy, which places women at risk of deficiency if nutritional dietary intake is not sufficient. Therapies involving iron modulation and regulation have important therapeutic usage across a spectrum of women’s health pathologies. In many gynecological conditions, an iron deficiency can develop, necessitating iron replacement. Conventional iron therapeutics have primarily centered around biological iron replacement without consideration of the consequences of iron overload. Modern therapeutic modalities target the byproducts of iron dysregulation, namely, free radical production often seen in endometriosis and vi gynecological cancers. Future therapies and diagnostics may see integration of hepcidin signaling, although their clinical usefulness has yet to be determined. The therapeutic regulation of iron at different levels may provide alternative treatment options for various gynecological pathologies.

## Figures and Tables

**Table 1:** Summary of iron related pathologies and therapeutics in Women’s health.

Condition	Mechanism of Iron Dysregulation	References
Cigarette Smoking during Pregnancy	Hypoxia induced suppression of hepcidin shifts iron to the bone marrow for ertheroposeis, transferring iron away from the fetus	[39,40,41]
Intrauterine Growth Restriction	Hepcidin induction by inflammation may reduce ferroportin expression on the placenta, impairing iron transfer	[42,43,44]
Preeclampsia	Excess iron early in pregnancy may affect spiral artery canulization. Hepcidin induction by inflammation may reduce ferroportin expression on the placenta, impairing iron transfer despite high circulating serum iron	[42,45]
Gestational Diabetes	Increased fetal iron transfer to fetus through an unknown mechanism	[46]
Macrosomia	Increased fetal iron transfer may increase growth	[47,48]
Uropathic *Escherichia coli*	Lysosomal release of ferritin bound iron increases propagation of *E. coli*	[49]
Bacteria Vaginosis	Iron deficiency may increase the risk of BV through an unknown mechanism	[50]
Abnormal Menstrual Bleeding	Increased blood loss because of abnormal menstrual bleeding depletes total body iron	[51]
Iron Deficiency and Iron Deficiency Anemia of Pregnancy	Increased fetal transfer of iron restricts available iron for erythropoiesis	[52,53]
Menopause	Cessation of menstruation increases total body iron, placing post-menopausal at risk for cardiovascular disease and cancer, through an unknown causal mechanism	[54,55,56,57,58,59]
Gynecological Anemia of Chronic Disease	Circulating inflammatory cytokines force systemic iron suppression	[60]
Endometriosis	Increased uptake of iron in endometriomas increases reactive oxygen species within the lesion, increasing release of inflammatory cytokines due to cell damage	[61,62,63,64]
Gynecological Cancer	Increased iron within gynecological malignancies leads to increased ROS mediated inflammation and mutagenesis, encouraging cancer growth	[65,66,67]

**Table 2:** Summary of iron related therapeutics in Women’s Health.

Iron Replacement	
Clinical Uses	Mechanism of Benefit
Abnormal Menstrual Bleeding	Direct iron repletion to offset menstrual blood loss
Iron Deficiency Anemia of Pregnancy	Replacement of Iron consumed from placental and fetal development
Gynecological Malignancy induced Anemia of Chronic Disease	Overcome immune mediated iron sequestration
Fatigue	Iron-depletion may explain fatigue without anemia in women. Mechanism of iron-depletion without anemia induced fatigue is unclear
Iron Chelation	
Clinical Uses	Mechanism of Benefit
Iron Overload of Pregnancy	Decrease iron toxicity in pregnancy induced iron overload in women receiving chronic transfusions for hereditary disorders
Proposed Clinical Uses	Proposed Mechanism of Benefit
Endometriosis	Melatonin and other drugs such as N-acteylcystenine may minimize abhorrent ROS species byproducts from localized iron dysregulation
	Delivery of miRNA targeting cellular iron handling can downregulate ROS production
Hepcidin	
Proposed Clinical Uses	Proposed Mechanism of Benefit
Diagnostic	Serum hepcidin concentration is correlated to multiple clinical disorders of interest in women’s health
Breast Cancer	Anti-Hepcidin therapies targeting the ferroportin receptor may prevent breast cancer growth
Ovarian Cancer and Breast Cancer	Hepcidin antagonists may inhibit the accumulation of high intracellular iron within gynecological malignancies, preventing cancer growth and improving prognosis
Urinary Tract Infection	Hepcidin agonists may further sequester iron from siderophilic colonizing bacteria, preventing bacterial growth
Endometriosis	Hepcidin antagonism may inhibit hepcidin mediated iron uptake
Intrauterine Growth Restriction	Hepcidin antagonism may inhibit hepcidin mediated suppression of placenta iron transfer to the fetus
Preeclampsia	High serum iron may contribute to disease pathogenesis, which may be reduced by hepcidin agonists
Other therapies	
Proposed Clinical Uses	Proposed Mechanism of Benefit
Ovarian Cancer	Transferrin-1 is overexpressed in ovarian carcinomas and anti-transferrin therapies may prevent cancer mediated uptake of iron

## Data Availability

All referenced material is published literature or organizational information.
